# Spike-timing dependent plasticity facilitates excitatory/inhibitory disbalances in early phases of tinnitus manifestation

**DOI:** 10.1186/1471-2202-13-S1-P1

**Published:** 2012-07-16

**Authors:** Christoph Metzner, Fabian Guth, Achim Schweikard, Bartosz Zurowski

**Affiliations:** 1 Institute for Robotics and Cognitive Systems, University of Luebeck, 23538 Luebeck, Germany; 2 Graduate School for Computing in Medicine and Life Sciences, University of Luebeck, 23538 Luebeck, Germany; 3 Department of Psychiatry, University Clinics Schleswig-Holstein, 23538 Luebeck, Germany

## 

The majority of tinnitus cases are related to cochlear dysfunction, leading to altered peripheral input to the central auditory system [1]. These alterations are believed to diminish the difference in activation during on- and off-conditions of sound [2]. As a compensatory means the affected region of primary auditory cortex tries to maximize the difference between basic level activity and sound-induced activity by changing the excitatory /inhibitory balance. In a previous model comprising ~3000 multi-compartment Hodgkin-Huxley-type neurons [3], we have shown that solely an increase of excitatory influences may be sufficient to achieve these maximization [3]. This previous Hodgkin-Huxley-type model [3] did not take into account synaptic plasticity, however.

Therefore we developed a simplified version where we could efficiently implement models of short-term and long-term synaptic plasticity. The structure and organization of the simulation was adopted from the Hodgkin-Huxley-type model [3], i.e. it consists of two groups of neurons, excitatory pyramidal cells and inhibitory basket cells in a 4:1 ratio, but with 71.875 neurons in total . The multi-compartment Hodgkin-Huxley-type model neurons were replaced by the simple neuron model proposed by Izhikevich [4]. Pyramidal cells were tuned to have a firing behaviour ranging from regular spiking to chattering, with a bias towards regular spiking, and basket cells were tuned to have fast spiking properties [5]. The neurons are equipped with glutamate- (AMPA and NMDA) and GABA-sensitive synaptic receptors having first-order linear kinetics [5]. Furthermore, we incorporated models of short-term depression (STD) and facilitation (STF) [6] as well as long-term synaptic plasticity [5]. The network consists of 8 clusters each of them coding a specific frequency, with one cluster resembling tinnitus-associated changes regarding noise and afferent input (TC, tinnitus cluster).

Importantly, the model largely showed the same results as the previous model, when we switched off all short- and long-term plasticity mechanisms. The tinnitus cluster shows an increased basic level of activity in the absence of input and, moreover, a diminished increase of activity during sound presentation of this particular frequency. Again a change of the ratio between excitatory and inhibitory weights towards more excitation and less inhibition in the TC results in a normal increase of activity during sound presentation.

Including the plasticity mechanisms results in an increase of excitatory weights over time in the TC. This results in a higher firing rate in TC and the tinnitus cluster shows an even higher basic level of activity during absence of sound which almost reaches the activity level during sound presentation before plastic changes. Furthermore, the 'pathological region' shows a dramatically increased synchronicity in firing behaviour which is also associated with tinnitus manifestation [1]. Further simulations showed that the long-term synaptic plasticity is the main driving force of these changes.

The presented simulation shows that synaptic plasticity facilitates processes that are believed to constitute the beginning of tinnitus manifestation. Our neurobiologically plausible simulation is an encouraging starting point for further modeling of the development of tinnitus, particularly due to its flexibility and computational efficiency.
